# (Dis)Agreement with Dysthanasia, Religiosity and Spiritual Experience as Factors Related to Nurses’ Workload during End-of-Life Care

**DOI:** 10.3390/ijerph20020955

**Published:** 2023-01-05

**Authors:** Brankica Juranić, Aleksandar Včev, Suzana Vuletić, Željko Rakošec, Domagoj Roguljić, Štefica Mikšić, Jelena Jakab, Jasenka Vujanić, Robert Lovrić

**Affiliations:** 1 Faculty of Medicine, Josip Juraj Strossmayer University of Osijek, 31000 Osijek, Croatia; 2 Department of Nursing and Palliative Medicine, Faculty of Dental Medicine and Health Osijek, Josip Juraj Strossmayer University of Osijek, 31000 Osijek, Croatia; 3 Clinic for Internal Diseases, University Hospital Centre Osijek, 31000 Osijek, Croatia; 4 Catholic Faculty of Theology in Đakovo, Josip Juraj Strossmayer University of Osijek, 31400 Đakovo, Croatia; 5 The Ministry of the Interior of the Republic of Croatia, 10000 Zagreb, Croatia

**Keywords:** dysthanasia, end-of-life care, intensive care units, psychological stress, religion, spirituality, workload

## Abstract

This study intended to investigate whether the workload of nurses in the course of providing end-of-life care correlated with their religiousness, spiritual experience and level of agreement with dysthanasia procedures. The respondents included 279 nurses from four Croatian hospitals. A structured and validated instrument was applied. Almost 90% of respondents are religious, and almost 45% of them have daily spiritual experiences. Respondents, especially those with high levels of religiousness and spiritual experience, express a low level of agreement with dysthanasia (mean = 58.21; score = 25–125). Moreover, nurses self-rated (on a scale of 1–5) their workload as quite high, especially when performing contradictory tasks imposed on them by their superiors (mean = 3.05) and during direct contact with dying patients and their family members (mean = 2.56). This significantly highest level of workload was experienced by the youngest nurses (*p* = 0.01) and nurses with little work experience (*p* < 0.01). This study also indicated that nurses who agree with dysthanasia experienced a higher level of workload when providing end-of-life care (r = 0.178; *p* < 0.01), while more frequent spiritual experiences reduced the level of workload (r = −0.205; *p* < 0.01). A deeper understanding of nurses’ attitudes toward dysthanasia, as well as of their religiousness and spiritual experiences, may ensure the collection of data beneficial to the timely identification of potential risks caused by workload.

## 1. Introduction

Rapid development of modern technology in all scientific disciplines, clinical medical sciences included, has resulted in increasingly intensified efforts to prolong life, both in healthy and seriously or terminally ill people. Therefore, the concept of dysthanasia has been more frequently researched and discussed in scientific and professional literature. Dysthanasia (from Greek: dys = bad, difficult; thanatos = death) is a relatively new concept developed within bioethics, and it refers to the application of medical procedures intended to maintain life at all costs, even when the condition is not curable, and medical procedures often prolong the patient’s pain and suffering [1]. Dysthanasia is associated with futile treatment and persistence with therapy, which do not benefit a patient but prolong the process of dying and not the patient’s life [1,2]. In this day and age, when technology is highly developed, complex and invasive medical procedures are frequently applied in the treatment of critical, life-threatening diseases, especially on modern hospital wards with sophisticated medical equipment (intensive care units, dialysis units, etc.) [1]. Despite the fact that the current Croatian Act on Protection of Patients’ Rights [3] clearly states that patients have the right to refuse medical procedures, physicians, nurses and other healthcare providers are often under pressure by patients’ families to perform all possible procedures to keep a patient alive at any cost. Numerous medical procedures are primarily directed to benefit the patients, but when providing end-of-life care (EoLC) to terminally ill and dying patients, they often include unnecessary physical and emotional pain [1,2,4,5,6]. In high-tech hospitals worldwide, dysthanasia procedures are becoming common and are performed when physicians, nurses and other healthcare providers are pressured by patients’ families to do whatever they can to keep the patients alive.

In compliance with the philosophy of high-tech diagnostic–therapeutic medicine, modern technology successfully solves medical problems, and death is considered a failure of medicine, not the inevitable end of human life [7]. However, death is a part of human life [8], and patients’ opinions about continuing their treatment should be respected. Respect for patients’ autonomy and their rights to decision-making are essential for delivering the best possible patient care, in which nurses have the key role. In these moments, a competent and experienced nurse is the person who can establish a dialogue between all members of a healthcare team and a patient’s family. Thus, nurses often find themselves in situations of bridging the communication gap between physicians and families of dying patients [9,10]. Nurses are there to ensure a humane approach and the safety of patients in situations in which bioethical principles and patients’ rights are often not considered [9]. Physicians often exclude nurses’ opinions and attitudes regarding decision-making and treatment [11]. However, taking into account that nurses are close to patients and their families, even though they are not responsible for a final decision, they have a vital role in this process, and they should be consulted by all other healthcare professionals [12]. When experienced nurses are respected and accepted by other members of a healthcare team, they are not afraid to offer their opinion and thus contribute to the decision-making process [13].

Caring for other people, as nurses typically do, is highly related to stress [14]. The risk of experiencing stress is especially high in nurses who provide EoLC, more specifically in nurses who work on wards, such as an intensive care unit (ICU), dialysis unit, oncology ward, etc., since there is a constant feeling of tension present [1,15,16]. The relevant literature describes numerous factors associated with challenges, difficulties and conflicts nurses encounter when providing EoLC: a high-tech clinical environment, complex nature of healthcare in dying patients, lack of professional experience, exposure to an acute and/or prolonged process of dying, interpersonal relationships within a healthcare team, communication and relationships with patients’ families, as well as nurses’ own physical and psychological fears and conditions caused by stressful situations [1,10,16,17]. Such demanding clinical obligations in providing for the various needs of dying patients cause significantly increased workload, stress and burnout, whereby nurses experience different physical, psychological and emotional signs of exhaustion and numerous symptoms and problems, such as headache, chronic fatigue, insomnia, nightmares, concentration difficulties, etc. [17,18].

The quality of healthcare provided to dying patients depends on nurses’ competencies, their beliefs, values and attitudes toward specific medical concepts, including dysthanasia, their spirituality, their satisfaction in the workplace, their stress level, their personality traits, their attitude towards dying and death, etc. [19]. Nurses’ spirituality is often associated with holistic (physical, psychological and spiritual), individualized and integrated care of dying patients [7,10]. Spiritual experience can be analyzed as a specific set of feelings, processes, or effects that occur in people in specific situations [20]. The word “spirituality” mainly refers to the deepest values and meanings by which people strive to live [20]. Thus, “spirituality” implies a vision of the human spirit and what will help it to fulfill its full potential. Through humanism, values and morality, spirituality is distinguished from all other things by its connection with the sacred and the transcendent. Spirituality is closely related to the supernatural and religion, although it extends beyond religion [20]. Spirituality can help nurses to understand that suffering makes sense in the transience of life and that being religious and spiritual play important roles in effective coping with emotions. Spiritual care is a vital factor in improving the quality of life of dying patients, members of their families or care providers [7,10]. Most studies have indicated a positive correlation between people’s spiritual experiences and their physical and psychical well-being, optimism and satisfaction with life, and a negative correlation between spirituality and psychosocial stress, anxiety and depression [10,21]. Nurses’ attitudes towards dysthanasia are different in different cultures and nations [22,23,24,25,26]. Since most societies are heterogeneous and have different approaches to the healthcare of dying patients, it has become necessary to conduct more comprehensive research focused on specific characteristics of previously mentioned approaches in different countries and cultures as well as on factors that influence nurses’ attitudes toward dysthanasia. The relevant literature has identified a number of individual and environmental stressors to which nurses who provide EoLC are exposed. However, it is evident there is a lack of studies that separately investigate and analyze correlations between nurses’ workload in the course of providing EoLC and their religious and spiritual aspects of life, as well as their level of agreement with dysthanasia procedures.

The objective of this study was to investigate the self-rated level of workload among nurses who provide EoLC for dying patients in the ICU and on other high-tech wards where EoLC procedures are performed. Furthermore, this study intended to investigate whether the workload of nurses in the course of providing EoLC correlated with their religiousness, spiritual experience and level of agreement with dysthanasia procedures.

## 2. Materials and Methods

### 2.1. Theoretical Framework

Due to different aspects of this study (psychological, physical, cognitive and spiritual), its origins and results cannot be based on only one theoretical framework. However, conceptual framework of this study is greatly based on Lazarus and Folkman’s transactional theory of stress and coping, according to which psychological consequences are caused by one’s own thoughts and understanding of situations [27]. Moreover, Folkman believes that psychological stress is caused by various threats that arise from person’s relationship with the environment. Another theory referring to spiritual aspects of this study, as well as aspects regarding human life and attitudes to dysthanasia, is certainly Frankl’s Theory of Meaning [28]. The three major concepts forming the basis of this theory are: a life purpose, freedom of choice and human suffering. These concepts are supported by three human dimensions: the physical or soma, the mental or psyche and the spiritual or noos. This theory tries to explain what impact providing EoLC to dying patients and the work environment have on all aspects of healthcare professionals’ quality of life (physical, psychological, spiritual, etc.). Moreover, the theory emphasizes the significance of specific areas and mechanisms used by nurses in palliative care to find the meaning of life through their own religiousness, spirituality and nature experiences [28].

### 2.2. Study Design

This cross-sectional study was conducted in the four largest hospitals in eastern Croatia, the European Union. The selection criteria for the healthcare institutions were: (a) a hospital has clinics/wards that provide EoLC to dying patients (ICU, dialysis unit and oncology ward); (b) a hospital is located in eastern Croatia (in order to gain insight into the investigated phenomenon in this region, bearing in mind that the researchers also come from this geographical area); (c) hospital administration issued the consent to conduct this research.

### 2.3. Respondents

The respondents included 279 licensed nurses at three levels of education: high school vocational education and training (VET), Bachelor of Science (BSc) and Master of Science (MSc). Selection criteria included: (a) respondents work in the ICU, dialysis unit and other clinics/departments/specialized wards that meet the previously mentioned criteria, (b) respondents provide direct EoLC to dying patients, (c) voluntary participation in the study. In order to be included in the study, respondents had to meet all the criteria.

The sample size was calculated using the online software program Sample Size Calculator by Creative Research Systems [29]. The calculation was based on the total number of nurses employed at the previously mentioned clinics/wards (321), with an initially defined confidence interval value of 3%, a confidence level of 95% and an alpha level of 0.05 [29]. According to calculations, the minimum sample size for this study was 247 respondents.

### 2.4. Instrument

The research instrument was a four-part questionnaire. The first part of the questionnaire included information on the socio-demographic characteristics of the respondents (age, gender, level of education, workplace, work experience, religious affiliation and religiousness).

The second part of the questionnaire was a validated instrument, the Questionnaire on Attitudes towards Dysthanasia and Patients’ Right to Co-decision (Q-ADPR) intended to investigate nurses’ level of agreement with dysthanasia [30]. The authors designed the original version of the questionnaire based on analysis of the relevant literature and extensive clinical experience in working with patients in the terminal stage of a disease [30]. The Q-ADPR consists of 25 items in total. The first eight items concern dysthanasia approaches and procedures when providing EoLC, while items 9–18 refer to the rights of dying patients to participate in decision-making during their treatment. The items 19–25 are formulated so that respondents play a hypothetical role of dying patients (“answer the following questions as if they refer to you personally, placing yourself into a role of a dying patient”). The items are formulated in this way so that the respondents give honest answers to questions regarding dysthanasia. Overall reliability of the Q-ADPR was 0.729, which indicated good reliability of this instrument. Factor analysis was conducted by the principal component method and resulted in one significant factor. The content validity of the Q-ADPR was analyzed and evaluated by a group of experts, which included a psychology professor, a methodologist, a statistics professor, two assistant professors with PhDs in nursing and two nurses with MScs in nursing and many years of experience working with oncology patients. The respondents’ answers were rated on a five-point Likert scale (Strongly Disagree = 1, Disagree = 2, Neither Agree nor Disagree = 3, Agree = 4, Strongly Agree = 5). The total score range was 25–125 points, whereby higher scores indicated a higher level of respondents’ agreement with dysthanasia. When calculating the total score, individual items were reverse-scored.

The third part of the questionnaire included the Daily Spiritual Experience Scale (DSES), the instrument to measure how respondents self-rated the frequency of their daily spiritual experiences when providing EoLC. The instrument was originally designed by Underwood and Teresi, based on comprehensive qualitative research [21]. The DSES is an instrument that was previously thoroughly psychometrically tested and validated in the Croatian context [20]. Psychometric analysis resulted in two related factors: the relationship with God and the relationship with others, which were also confirmed in this study. The reliability of the Croatian version of the DSES scale was tested using Cronbach’s alpha coefficient. Internal consistency reliability for the complete questionnaire in this study was 0.95 (0.94 for the first factor and 0.85 for the second factor). The questionnaire consists of 16 items in total. The respondents were asked to rate the frequency of their own spiritual experiences on a 6-point Likert scale, where in the first 15 items, 1 was Never or almost never and 6 was Many times a day, while in the last item, they were asked to self-rate the level of their own closeness to God on a 4-point Likert scale, where 1 was Not close and 4 was As close as possible. The total score range was 16–94 points, where a higher score indicated more frequent spiritual experiences and feeling closer to God.

The fourth part of the questionnaire was a seven-item scale where respondents self-rated their experienced workload while providing EoLC. The items were formulated based on research of the relevant literature and the extensive experience of the researchers (the authors of this article) in working with dying patients in terminal stages of diseases. Moreover, preliminary talks with 20 nurses who provide care for dying patients contributed to the formulation of the seven items. These talks were focused on their experience, challenges, difficulties, reasons for work overload and stress while providing EoLC. The content validity of the items was confirmed by the expert committee, which included two psychology professors, a methodologist, a statistics professor, two professors with PhDs in nursing and two nurses with MScs in nursing and many years of experience working with dying patients. After the content validation and proofreading of the grouped items, their precision and clarity were tested by a number of randomly selected volunteers [31]. The volunteers were nurses selected by the same method as the respondents [31]. After they had checked the clarity and comprehensibility of the items, they gave researchers their feedback but did not participate in the research as respondents. The internal consistency and reliability of the scale regarding respondents’ workload was 0.781, which indicated good reliability. The respondents were asked to rate their level of agreement on a 5-point Likert scale (1 = Strongly disagree and 5 = Strongly agree). The total score range was 7–35 points, where a higher score indicated a higher level of experienced workload in nurses.

### 2.5. Data Collection

The data were collected over a four-month period in intensive care units (ICU), dialysis units and oncology wards at four healthcare institutions included in the research. The researchers (the authors of this article) distributed the printed questionnaires to the nurses, who filled them in using a pen. The respondents filled in the questionnaires during a break on their wards, using the conference room. The completion of the questionnaire took, on average, 20 min and was not time-limited. During the research period, a total of 321 nurses were employed on the mentioned wards. They were all offered to participate in the research, and 283 nurses were interested in filling in the questionnaire, which was 88.2% of all nurses. During further analysis, four anonymous questionnaires were excluded, since they were not completely filled in. Thus, a total of 279 questionnaires were analyzed.

### 2.6. Data Analysis

Descriptive statistics were used for nominal variables, and the data are presented as proportions and percentages. Numerical data are described using the basic measures of mean and dispersion (arithmetic mean and standard deviation). The Shapiro–Wilk test was used to calculate the normality of the distribution of numerical variables. The Student *t*-test was used to determine the difference between measured parameters in two groups (parametric distribution). The parametric test and one-way analysis of variance test (ANOVA) were used to compare mean differences between two independent groups (applying the Bonferonni post-hoc test). The Pearson correlation coefficient was used to measure the correlation between individual parameters. The statistical analysis of the reliability of the instrument was tested using Cronbach’s alpha coefficient. The significance level for the obtained results was set as α = 0.05. SPSS for Windows software (version 22.0, IBM SPSS, Armonk, NY, USA) was used for data analysis.

### 2.7. Ethical Considerations

The respondents were informed of all the details and ethical considerations regarding this study prior to filling in the questionnaire. They were guaranteed anonymity, both during filling in the questionnaire and after the research, and the data obtained during the research were available only to the researchers. The respondents had the right to drop out of the study at any moment. Only the researchers had access to research data. The researchers had obtained the author’s consent to use the original version of the Daily Spiritual Experience Scale (DSES) for translation and as a research instrument in this study. The study was approved by the relevant Ethics Committee (IRB approval number 2158-61-07-17-05).

## 3. Results

### 3.1. Sociodemographic Characteristics of Respondents

The research included 279 nurses in total, out of whom 49 (17.6%) were men and 230 (82.4%) were women. The median age was 39 (interquartile range: 31–49 years of age). The majority of them, 155 (55.6%), work in the intensive care unit. Regarding the level of education, there were 163 (58.4%) respondents with completed vocational education and training for licensed nurses. Regarding their religious affiliation, 251 (90%) respondents were Catholics, while the other religions existed in small numbers (Table 1).

### 3.2. Respondents’ Level of Agreement with Dysthanasia

The total mean value of the scale regarding respondents’ level of agreement with dysthanasia was 58.21 (SD = 3.89) (Table 2). Regarding the subscales, the lowest level of agreement (arithmetic mean = 20.5; SD = 3.78) was expressed for the subscale “Dysthanasia procedures in hypothetical role of dying patients”, while the highest level of agreement and support was expressed for the subscale “The rights of dying patients to participate in decision-making regarding their treatment” (Table 2).

The results indicated significant differences regarding the respondent’s level of agreement with some of the key items on dysthanasia. The item “*Life sustaining treatment of a patient, whereby extraordinary measures are permanently applied, is justified even when there is no hope of cure*” in the subscale “Dysthanasia procedures during EoLC”, was not agreed with by 138 respondents (49.5%), while 96 (34%) neither agreed nor disagreed and only 46 respondents (16%) agreed with the item. As many as 266 respondents (95%) agreed with the item “*The treatment goal in dying patients should be to relieve their suffering and pain and not to prolong life at any cost*”, while only nine (3%) disagreed with it. Furthermore, 181 respondents (65%) agreed with the item “*Pointless medical procedure has little or no benefit for a patient, but increases the suffering of both patients and their families*”, while 67 respondents (24%) neither agreed nor disagreed and only 31 (11%) did not agree with the item.

In the subscale “The rights of dying patients to participate in decision-making regarding the course of treatment”, 266 respondents (95%) agreed with the item “*Patients should have the right to refuse or accept a procedure, being informed and explained they have a free choice*”. The item “*The right to participate in decision-making is the essence of informed consent*”, was agreed with by 228 respondents (81.7%), while 40 respondents (14%) neither agreed nor disagreed and only 11 (4%) disagreed with the item.

In the subscale “Dysthanasia procedures during EoLC (respondents in the hypothetical role of dying patients)”, 228 respondents (82%) agreed with the item “*I think that quality of life has advantage over prolonged life*”, while only 16 respondents (5.7%) disagreed. In addition, 192 respondents (81.7%) agreed with the item “*I do not support enormous efforts and useless means that help no one and only slightly prolong dying process, causing additional strain for all people involved in the proces*s”, while 56 respondents (20%) neither agreed nor disagreed and 31 (11%) disagreed.

### 3.3. Respondents’ Daily Spiritual Experiences

The total mean value of the DSES scale regarding the frequency of daily spiritual experiences when providing EoLC was 55.41 (SD = 0.92) (Table 3). Almost 45% of respondents stated that as far as the 15 items about spiritual experience are concerned, they experience them on a daily basis. According to the mean values for their answers, the highest spiritual experience frequency (arithmetic mean = 3.92) was expressed for the item “*I feel thankful for my blessings*”, while the least frequent spiritual experience was expressed for the item “*During worship, or at other times when connecting with God, I feel joy which lifts me out of my daily concerns*”. As for the last item, concerning closeness to God, as many as 154 respondents (55%) stated they feel very close or as close as possible to God (Table 3).

### 3.4. Nurses’ Workload When Providing EoLC

The total mean value for the scale of respondents’ workload when providing EoLC was 17.75 (SD = 3.45). According to mean values for the items, the highest level of workload (arithmetic mean = 3.06) was expressed for performing contradictory tasks imposed on them by their superiors, while the lowest workload (arithmetic mean = 2.22) was experienced when patients considered them persons of trust.

The results obtained by analysis of the answers regarding respondents’ workload when providing EoLC indicated that 125 respondents (55.1%) agreed and a further 38 (16.7%) strongly agreed they had the required knowledge and skills to work with patients who need end-of-life care (Table 4). As many as 110 respondents (48.5%) agreed and 13 (5.7%) strongly agreed that in caring for patients at the end of life, some interventions may be performed in a different way. In the item regarding performing contradictory tasks imposed on them by the superiors, 81 (35.7%) respondents chose the option neither agree nor disagree, which was the largest number of respondents who chose this answer. As many as 102 respondents (44.9%) agreed and a further 17 (7.5%) strongly agreed that they often perform tasks for which they do not have enough vital data and information. Moreover, 140 respondents (61.7%) agreed and 21 (9.3%) strongly agreed with the item stating that patients in their last days considered the respondents to be persons of trust.

Finally, 100 respondents (44.1%) agreed and 35 (15.4%) strongly agreed that direct contact with patients in their last days and their family members is too stressful (Table 4).

Differences in respondents’ workload when providing EoLC were analyzed according to respondents’ general characteristics, and the obtained results indicated that the level of workload was significantly higher in the group of the youngest respondents, in the age range of 18–25 years (arithmetic mean = 19.7; SD = 3.61; *p* = 0.01) (Table 5). Moreover, the significantly highest level of workload was expressed by respondents with the least work experience, ranging from 1–5 years (arithmetic mean = 19.4; SD = 3.26; *p* < 0.01).

According to other respondents’ characteristics (gender, level of education, workplace and religious affiliation), there were no significant differences in the level of workload (Table 5).

### 3.5. Correlation between Respondents’ Level of Workload and Their Agreement with Dysthanasia, Religiousness and Spiritual Experience

The results showed a significant correlation between respondents’ level of workload when providing EoLC and their agreement with dysthanasia (r = 0.178; *p* < 0.01) and a significant negative correlation between workload and their level of daily spiritual experience (r = −0.205; *p* < 0.01). Spiritual experience also significantly correlated with self-rated level of religiousness (r = 0.639; *p* < 0.01) (Table 6).

## 4. Discussion

This study sought to provide deeper insight into nurses’ workload when providing EoLC, as well as possible correlations between workload and their religiousness, spiritual experiences and their level of agreement with dysthanasia.

### 4.1. Respondents’ Agreement with Dysthanasia

The results of this study showed that 90% of respondents consider themselves religious, and 90% are declared Catholics, which was expected. According to the last census carried out in 2021, 78.97% of the population in the Republic of Croatia declared themselves as Catholics, and around 5% declared belonging to other religious affiliation, whereas only 4.71% of the population declared having no religious affiliation [32]. The Republic of Croatia and Poland are the countries with the largest percentage of population declared as Catholics among all Slavic countries [32].

The nurses who participated in this study generally disagreed with dysthanasia procedures when providing EoLC, which is in compliance with the results of similar studies [33]. This is supported by the obtained mean value of 58.21 (range 25–125), since a higher score indicates a higher level of agreement with dysthanasia. The results of the study by Rostami et al. [25] indicated that the majority of nurses (65.7%) had a moderate perception of futile care, which is also supported by another study [34].

Moreover, the results of our study, obtained in subscales and in some respondents’ answers, indicated the respondents’ low level of agreement with dysthanasia. Thus, the item “Life sustaining treatment of a patient, whereby extraordinary measures are permanently applied, is justified even when there is no hope of cure”, was disagreed with by 49.5% of respondents, while only 16% agreed with it. Furthermore, as many as 95% of respondents agreed with the item “The treatment goal in dying patients should be to relieve their suffering and pain and not to prolong life at any cost”, while only 3% disagreed with it. These results clearly indicate that a high percentage of nurses disagree with dysthanasia procedures when providing EoLC. When the respondents were given the hypothetical role of dying patients in the third part of the questionnaire, they disagreed with dysthanasia procedures in almost all items or chose the option Neither Agree nor Disagree. For instance, as many as 82% of respondents agreed with the item stating that quality of life has advantage over prolonging their life, while only 5.7% disagreed with it. It is evident that the majority of respondents respect their own moral autonomy and do not expect their lives to be prolonged by pointless medical treatments. Their attitude toward dysthanasia is clearly reflected in their own wishes and expectations regarding themselves. In the study by Silva [35], the nurses-respondents expressed a similar opinion, additionally emphasizing the need to open a constructive discussion among all healthcare professionals and dying patients in the process of EoLC, based on the principle of respect for patients’ autonomy [35].

Taking into account that all nurses participating in this study provide EoLC on a daily basis on high-tech wards, such as ICU, where invasive medical procedures are frequently applied, it is justified to express concern, since nurses evidently witness and/or directly carry out certain care procedures that are not in line with their beliefs, values and attitudes. According to Rostamy et al. [25], nurses providing EoLC often perform actions that they consider futile but must carry them out at all costs. Such and similar situations can significantly disrupt their perception of situations in the clinical environment. It is important to remember that the quality of the EoLC provided is influenced by nurses’ opinions and their attitudes toward death and dying [10,19]. Moreover, nurses’ presence at the end of a patient’s life is vital to recognize situations in which bioethical principles and the patient’s rights are not taken into account [7]. Health promotion and bioethics have the common goal of respecting human dignity. Dying with dignity is the final outcome of a life lived with dignity. From the perspective of dying with dignity, nurses are crucial for maintaining a patient’s dignity [7].

Furthermore, 95% of respondents in this study agreed that a patient should have the right to refuse or accept a procedure after being informed about it and explained about having free choice. This indicates that almost all respondents understand and agree with involving patients in decision-making and respecting their wishes during EoLC. However, healthcare professionals in ICUs and other wards conducting EoLC are frequently under constant pressure applied by the patient’s family to take all possible measures in order to keep a patient alive [5]. Preserving life at any cost has become a commonly accepted dogma in high-tech wards worldwide.

### 4.2. Daily Spiritual Experiences of Respondents

Considering the total mean value of the DSES scale of 55.41 (range 16–94), the level of daily spiritual experience may seem moderately high. However, the analysis of the items on the scale clearly indicates that the majority of respondents declared for all 15 items that they have daily spiritual experiences. This result shows that having a spiritual experience is significantly more frequent in respondents when providing EoLC. The previously mentioned total mean value of the DSES scale is most probably the result/consequence of a wide range on a 6-point frequency scale when it comes to a significant distribution/dispersion of respondents’ answers on the scale. As many as 154 respondents (55%) declared that they feel close to God or as close as possible, which also suggests a high level of their spirituality. This result was actually expected, since almost 90% of respondents declared they were religious. In the Christian tradition of western civilizations, it is impossible to separate religiousness and spirituality completely [36]. Religiousness refers to a personal or individual relationship with God, that is, a person’s ability to communicate with God. Spirituality, however, refers to the area of human existence that lies in the transcendent nature of all matter, which gives one’s life the feeling of meaningfulness, interconnection, integrity and hope. Spirituality includes religiousness, and people who formally do not belong to any religion also have spiritual needs [36].

The nature and course of EoLC greatly depend on a patient’s condition and the personal perspective of healthcare professionals, whereas nurses’ attitudes to dysthanasia significantly depend on their personal judgements that arise from their feelings, personal beliefs, their religion and spirituality, as well as their social and cultural environment [37]. Spirituality is a crucial aspect of caring for dying patients, whose goal is to integrate physical, psychological and spiritual care for patients and to maintain the integrity of both patients and their families [10,32].

### 4.3. Nurses’ Workload When Providing EoLC

The nursing profession is generally associated with a high level of workload and work-related stress, especially for nurses who provide EoLC [5,7,8,14,15,16,38]. The results obtained in this study indicated that the total mean value of the nurses’ workload scale was 17.75 (range 7–35). These results are in compliance with the results of a study recently carried out in Portugal, where nurses expressed a moderate level of workplace stress that caused various personal difficulties and consequences, which influenced the quality and safety of the care provided [5]. In our study, nurses experienced the highest level of workload in situations when they needed to perform contradictory tasks imposed on them by their superiors and when they needed to perform tasks for which they did not have enough vital data and information, which is nowadays recognized as a problem nurses encounter in their work [39,40]. In their study, Sarafis et al. [40] reported disagreements and problems with superiors as a third major stressor in nurses. The imposition of contradictory tasks was a cause of stress for 26% of respondents in this study, and 35% of respondents did not agree or disagree, which usually implies that a certain percent of respondents from this group had similar experiences. Another intensive stressor, identified by 43% of respondents, was frequently performing tasks for which they did not have enough vital data and information. This is a frequently stated and well-known challenge and problem that can cause a feeling of inadequate competence and efficiency within a healthcare team [9,41]. Moreover, a significant stressor for 44% of respondents was awareness of the fact that certain interventions can be performed in a different way in patient’s last days. The option Neither agree nor disagree was chosen by 35% of respondents, which implies the possibility of an even greater incidence of this stressor. This result indicates the fact that nurses still have a secondary role when decisions about patients are made within a healthcare team [11]. Furthermore, as many as 49% of respondents felt stress because of direct contact with dying patients and their family members, which is completely in compliance with other studies and relevant literature, where this very relation is recognized as a very common stressor and cause of burnout syndrome in nurses [5,14,15,16,17,42,43,44]. Moreover, the high level of nurses’ workload was caused by the fact that patients in their last days considered them to be persons who could solve all their vital problems, while nurses are aware that they cannot do that, which results in considerable stress. This is a very well-known stressor when providing EoLC, whereby nurses often feel they are not competent enough for their work, which leads to further frustrations and negative consequences [5,18].

The results of this study also showed that 60% of respondents thought they had the required knowledge and skills to work with patients who needed end-of-life care and 58% thought that patients considered them persons of trust, which resulted in the lowest level of stress in respondents, as expected. However, despite all this, there were still 7.5% of respondents who thought they did not have the required competence for providing EoLC, and 18.9% neither agreed nor disagreed with this item. These percentages might not seem high, but they need to be seriously considered since all the respondents work on high-tech wards with high-risk patients. Therefore, it is of utmost importance to establish a system that can ensure various methods of both formal and informal education of nurses at all levels of education with a focus on EoLC. It is necessary to develop active educational-intervention strategies to improve all types of communication skills and resolve intradisciplinary and interdisciplinary conflicts, to improve strategies to manage stressful situations, to cope with one’s own difficulties regarding the process of dying and death, spiritual needs, managing complex situations in the course of providing EoLC and to establish quality relationships between nurses and patients at the end of their lives as well as with their families [5].

### 4.4. Correlation between Respondents’ Workload and Their Agreement with Dysthanasia, Religiousness and Spiritual Experience

The results of this study showed there were correlations between nurses’ self-rated workload and various socio-demographic characteristics, their level of agreement with dysthanasia, their religiousness and their spiritual experience. There were significant differences in the nurses’ workload in the course of providing EoLC according to their age. The youngest respondents, in the age range 18–25, as well as respondents with the least work experience, self-rated their workload at the highest level, which is in compliance with the results of similar studies [2,5,45]. In their study, Costeira et al. [5] reported that novice nurses with little experience in providing EoLC experienced a higher level of stress, especially in relation to exposure to the process of dying and death. Other studies also report that experienced nurses have a lower level of workload and apply more efficient strategies of stress management [2,45]. As opposed to that, one study conducted in Iran did not find any correlation between age and nurses’ workload [46]. It is possible that older nurses with more work experience who participated in that study also had more efficient mechanisms of coping with specific stressors when providing EoLC. Such coping mechanisms are usually based on previously experienced exposure to the prolonged process of dying and the challenges of EoLC, along with more skills and experience of working in a healthcare team and with patients’ families. Furthermore, the results of another recently conducted study [39], where the objective was to investigate the level of workload in nurses employed in two hospitals, indicated that dying and death were considered major stressors. However, the same study also reported contradictory results in relation to our study, since the respondents younger than 25 years of age expressed a significantly lower level of stress in comparison with older respondents, especially those older than 50 years of age. The authors of that study [39] explained their results by the fact that the oldest respondents with the longest work experience were exposed to stressors for too long, which resulted in their burnout. This conclusion is supported by other authors as well [47]. These conclusions clearly suggest that the lower and upper limits of the age range are the key factor for the level of workload, since the highest stress levels are experienced by both youngest and least experienced nurses as well as those exposed to major stressors when providing EoLC for a long time. Considering other socio-demographic characteristics of respondents (gender, level of education, workplace, marital status and religious affiliation), there were no significant differences in respondents’ level of workload in this study, while in some other studies there were significant differences in respondents’ workload according to gender [39] and marital status [48].

The results of this study showed there was a significant correlation between the nurses’ self-rated level of workload when providing EoLC and their level of agreement with dysthanasia. According to this study, nurses who expressed a higher level of agreement with dysthanasia procedures also simultaneously expressed a significantly higher level of workload. These results were somewhat unexpected, since it was logical to assume that nurses who do not agree with dysthanasia procedures would experience a higher workload while providing them. However, this unexpected but simultaneously not very surprising result is probably a reflection of the typical contemporary high-tech approach to patients’ treatment, especially taking into account the workplaces of the respondents in this study (ICUs, dialysis units and oncology wards). It is well known that in nursing practice there is a difference between humane care models, which is the model most nurses have chosen their profession for, and the so-called biomedical model, which is primarily oriented to task performance and reflects the reality of institutional demands but places nurses in a subordinate role in which they receive physicians’ orders and follow them [9]. Such a role, where nurses only follow orders, and are not allowed to act autonomously, meaning that care and caring relationships that enable establishing connection with a patient often take second place [9,11]. This is especially evident on wards such as ICU, but also on all other wards where nurses provide EoLC. Nurses’ inclination to accept such a role and to start thinking in this way, especially in ethically sensitive situations, is worrying but at the same time not surprising, considering the current situation in the global healthcare system. Facing the increased workload, both in terms of quantity and complexity, and a lack of certainty and understanding, nurses tend to seek something to hold onto in order to reduce anxiety related to uncertainty and insecurity and to survive in a complex, high-tech healthcare system [9]. Therefore, adaptability is a suitable way of functioning and facing these circumstances. Rules, norms, standards and protocols offer the solution for nurses to simplify and control complex situations, which leads to a false sense of security but can also be an additional source of dissatisfaction for nurses and result in an increased level of stress [9]. One comparative study describes interesting testimonies of nurses who left their workplace due to burnout. They stated that during the performance of certain tasks, they often had to oppose their own conscience and sometimes “deaden” it in order to do the work expected of them in the modern healthcare system [49]. It is therefore not surprising that nurses, over time, have developed such an approach, since it is often desirable in the contemporary world. Such an explanation may indicate the fact that applying the ethics of care requires nurses to not only have competence and compassion but also courage. Establishing relationships with dying patients while providing care and behaving according to one’s own values and beliefs, which are not in line with current expectations and regulations, is not always safe, and it is a real challenge for nurses [9]. At any rate, there are a number of questions about what personal, professional and situational factors contribute to nurses’ ways of thinking and attitudes toward providing EoLC, especially in sensitive ethical aspects. Nurses’ attitudes are formed based on available (ir)relevant information, their knowledge, beliefs, values and experiences. Some studies indicate that there is a lack of knowledge and understanding of ethical terms and concepts among nurses working in ICUs and other wards where EoLC is provided [1]. All of the above-mentioned information confirms the evident need for more comprehensive communication and discussion with nurses, putting emphasis on different attitudes toward various ethical concepts in their practice.

In this study, there was a significant negative correlation between respondents’ self-rated workload and the frequency of their spiritual experiences, whereby nurses with more frequent spiritual experiences expressed a lower level of workload. These results are not surprising, since a person’s spiritual experience is related to satisfaction with life and a lower level of anxiety and psychosocial stress, according to available literature [10,20]. Moreover, a small percentage of respondents with frequent spiritual experiences agree with dysthanasia, which is probably in compliance with their religious belief that the ethical goal of caring for dying patients is to make their process of dying easier and not to prolong their suffering [7]. At any rate, let us not forget that high percentage of respondents in this study declared being religious. The results of other studies also confirmed that there was a significantly lower level of work-related stress in nurses with a higher level of spiritual experience [21]. It is also important to remember that in Christian tradition, it is impossible to completely separate spirituality from religiousness [37], which is supported by the results of this study, which indicated a significantly high correlation between respondents’ self-rated level of religiousness and their daily spiritual experience. As described earlier, spirituality helps nurses understand that a person’s suffering makes sense in the transience of life, and religiousness and spirituality contribute to efficient coping with emotions [7,10].

### 4.5. Limitations of the Study

There are certain limitations to this study. To start with, it is a cross-sectional quantitative correlational study that does not have a (quasi)experimental approach and cannot determine a direct causal relationship between the variables in the research. Thus, the study does not have causal properties. Furthermore, considering the data collection in this study (questionnaire survey) there is a possibility that the respondents gave socially desirable answers, despite the fact that the questionnaire is completely anonymous. Finally, the respondents’ answers at the moment of filling in the questionnaire during their working hours may be influenced by acute swings in their mood, which are characteristic for nurses when providing EoLC, depending on the current work atmosphere and relationships in the clinical environment.

## 5. Conclusions

Our study confirmed that nurses who provide EoLC, and especially those with high levels of religiousness and spiritual experience, mostly disagree with dysthanasia. Moreover, nurses self-rated their workload when providing EoLC as significantly high, especially when performing contradictory tasks imposed on them by their superiors and during direct contact with dying patients and their family members. The highest level of workload was experienced by the youngest nurses with little work experience. This study also indicated that nurses who agreed with dysthanasia experienced a higher level of workload when providing EoLC, while more frequent spiritual experiences reduced the level of workload. Agreement with dysthanasia and, at the same time, the higher level of workload in nurses are probably the consequences of the currently dominant biomedical model, along with the omnipresent philosophy of continuing life-sustaining treatment of dying patients. The accumulation of difficult tasks and feelings of misunderstanding and insecurity related to their work force nurses to adapt and accept the imposed way of thinking and working. This often contradicts fundamental humane models of nursing care, which additionally intensify nurses’ frustrations and workload. Therefore, a deeper understanding of nurses’ attitudes toward dysthanasia as well as of their religiousness and spiritual experiences may ensure the collection of data beneficial to the timely identification of potential risks caused by workload. Moreover, the obtained data can be a useful base to plan strategies and methods to prevent nurses’ workload when providing EoLC in high-tech and extremely demanding clinical environments (improved working conditions, supervisory groups, formal and informal educational programs related to EoLC, lectures and discussions focused on ethical dilemmas, communication skills training, cognitive-therapy interventions, relaxation interventions and informal socializing of healthcare teams).

## Figures and Tables

**Table 1:** Socio-demographic Characteristics of Respondents (n = 279).

Gender	
men	49 (17.6)
women	230 (82.4)
Workplace	
ICU	155 (55.6)
dialysis unit	56 (20.1)
oncology ward	68 (24.4)
Work experience	
0–5	43 (15.4)
6–10	33 (11.8)
11–20	71 (25.4)
21–30	85 (30.5)
more than 30	47 (16.8)
Level of education	
VET	163 (58.4)
BSc	114 (40.9)
MSc	2 (0.4)
Marital status	
single	66 (23.7)
married	183 (65.6)
divorced	24 (8.6)
widowed	3 (1.1)
common-law marriage	3 (1.1)
Religious affiliation	
Catholic	251 (90)
Orthodox	10 (3.6)
Muslim	2 (0.7)
atheist	5 (1.8)
agnostic	5 (1.8)
other	6 (2.2)
Self-rated religiousness	
very religious	85 (30.5)
mostly yes	90 (32.3)
moderately	80 (28.7)
mostly not	8 (2.9)
not at all	16 (5.7)
Total	279 (100)

**Table 2:** Nurses’ level of agreement with dysthanasia (n = 279).

Q-ADPR Subscales		Level of Agreement with Dysthanasia		
		Number of Items	Range of Points *	Arithmetic Mean (SD)
1.	Dysthanasia procedures when providing EoLC	8	(8–40)	20.51 (3.78)
2.	The rights of dying patients to participate in decision-making regarding the course of treatment	10	(10–50)	22.23 (3.44)
3.	Dysthanasia procedures during EoLC (respondents in the hypothetical role of dying patients)	7	(7–35)	15.51 (4.19)
Total Q-ADPR score		25	(25–125)	58.21 (3.89)

* higher score = higher level of agreement with dysthanasia.

**Table 3:** Nurses’ daily spiritual experiences when providing EoLC (n = 279).

DSES Scale Items	Spiritual Experience Frequency	
	Range of Points *	Arithmetic Mean (SD)
1. I feel God’s presence.	1–6	3.53 (1.29)
2. I experience a connection to all of life.	1–6	3.61 (1.12)
3. During worship, or at other times when connecting with God, I feel joy which lifts me out of my daily concerns.	1–6	2.85 (1.31)
4. I find strength in my religion or spirituality.	1–6	3.53 (1.13)
5. I find comfort in my religion or spirituality.	1–6	3.54 (1.10)
6. I feel deep inner peace or harmony.	1–6	3.37 (1.12)
7. I ask for God’s help in the midst of daily activities.	1–6	3.67 (1.23)
8. I feel guided by God in the midst of daily activities.	1–6	3.62 (1.16)
9. I feel God’s love for me directly.	1–6	3.46 (1.28)
10. I feel God’s love for me through others.	1–6	3.32 (1.25)
11. I am spiritually touched by the beauty of creation.	1–6	3.39 (1.17)
12. I feel thankful for my blessings.	1–6	3.92 (0.98)
13. I feel a selfless caring for others.	1–6	3.79 (0.91)
14. I accept others even when they do things I think are wrong.	1–6	3.55 (1.12)
15. I desire to be closer to God or in union with the divine.	1–6	3.33 (1.20)
16. In general, how close do you feel to God?	1–4	2.93 (0.94)
Total DSES scale	16–94	55.41 (3.24)

* higher score = higher frequency of spiritual experiences and higher level of closeness to God.

**Table 4:** Nurses’ level of workload when providing EoLC-distribution of respondents’ answers (n = 279).

Items		Respondents Answers n (%)					Points	
		Strongly Disagree	Disagree	Neither Agree Nor Disagree	Agree	Strongly Agree	Range of Points *	Arithmetic Mean (SD)
1.	I have required knowledge and skills to work with patients who need end of life care.	1 (0.4)	20 (8.8)	43 (18.9)	125 (55.1)	38 (16.7)	1–5	2.27 (0.88)
2.	In patients at the end of life some interventions may be performed in a different way.	5 (2.2)	25 (11.0)	74 (32.6)	110 (48.5)	13 (5.7)	1–5	2.55 (0.83)
3.	In my work with patients in their last days, my superiors often impose performing contradictory task.	7 (3.1)	66 (29.1)	81 (35.7)	61 (26.9)	12 (5.3)	1–5	3.06 (0.96)
4.	I often perform tasks for which I do not have enough vital data and information.	3 (1.3)	37 (16.3)	68 (30.0)	102 (44.9)	17 (7.5)	1–5	2.56 (0.90)
5.	Patients in their last days consider me as a person who will solve some of their vital problems.	0 (0)	31 (13.7)	91 (40.1)	92 (40.5)	13 (5.7)	1–5	2.45 (0.78)
6.	Patients in their last days consider me as a person of trust.	1 (0.4)	6 (2.6)	59 (26.0)	140 (61.7)	21 (9.3)	1–5	2.22 (0.64)
7.	Direct contact with patients in their last days and their family members is too stressful for me.	6 (2.6)	29 (12.8)	57 (25.1)	100 (44.1)	35 (15.4)	1–5	2.49 (1.01)
Total score in the workload scale							7–35	17.75 (3.45)

* higher mean value = higher level of workload.

**Table 5:** Differences in respondents’ level of workload according to general characteristics (n = 279).

Respondents’ Characteristics		Level of Workload	
		Arithmetic Mean (SD) (Range of Points 7–35) *	*p* Value
Gender	male	18.1 (3.89)	0.42 **
	female	17.7 (3.02)	
Age (years)	18–25	19.7 (3.61)	0.01 ^†^
	26–35	17.9 (2.86)	
	36–45	17.2 (3.20)	
	>50	17.6 (3.13)	
Level of Education	VET	17.7 (3.26)	0.28 ^†^
	BSc	17.8 (3.07)	
	MSc	20 (2.92)	
Workplace	ICU	17.9 (3.24)	0.36 ^†^
	dialysis unit	17.2 (2.99)	
	oncology ward	17.7 (3.19)	
Work experience (years)	0–5	19.4 (3.26)	<0.01 ^†^
	6–10	17 (3.19)	
	11–20	17.7 (2.76)	
	21–30	17.1 (3.23)	
	>30	17.8 (3.18)	
Marital status	single	18.5 (3.14)	0.16 ^†^
	married	17.5 (3.10)	
	divorced	17.6 (3.33)	
	widowed	17.7 (5.86)	
	common-law marriage	20 (4.58)	
Religious affiliation	Catholic	17.8 (3.17)	0.23 ^†^
	Orthodox	18.7 (2.65)	
	Muslim	17 (4.24)	
	atheist	15.6 (1.52)	
	agnostic	19 (1.00)	
	other	15.5 (5.39)	

* higher score = higher workload; ** Student’s *t*-test; ^†^ one-way analysis of variance.

**Table 6:** Correlation between nurses’ workload when providing EoLC and their attitude toward dysthanasia, religiousness and spiritual experience.

	Religiousness	Agreement with Dysthanasia	Spiritual Experience	Workload When Providing EoLC
Religiousness	-			
Agreement with dysthanasia	−0.064	-		
Spiritual experience	0.639 *	−0.057	-	
Workload when providing EoLC	0.083	0.178 *	−0.205 *	-

* *p* < 0.01.

## Data Availability

All data generated analyzed during the current study are available from the corresponding author on reasonable request.
